# Graphene-Doped Tin Oxide Nanofibers and Nanoribbons as Gas Sensors to Detect Biomarkers of Different Diseases through the Breath

**DOI:** 10.3390/s20247223

**Published:** 2020-12-17

**Authors:** Carlos Sánchez-Vicente, José Pedro Santos, Jesús Lozano, Isabel Sayago, José Luis Sanjurjo, Alfredo Azabal, Santiago Ruiz-Valdepeñas

**Affiliations:** 1 Institute of Physics Technology and Information (CSIC), 28006 Madrid, Spain; jp.santos@csic.es (J.P.S.); i.sayago@csic.es (I.S.); jl.s.m.3@csic.es (J.L.S.); 2 Up Devices and Technologies, 28021 Madrid, Spain; alfredo@updevices.com (A.A.); santiago@updevices.com (S.R.-V.); 3 Industrial Engineering School, University of Extremadura, 06006 Badajoz, Spain; jesuslozano@unex.es

**Keywords:** electronic nose, nanosensor, nanofibers, nanoribbons, nanostructures, gas sensors, biomarkers, graphene, respiratory diseases, digestive diseases, chronic diseases, volatile organic compounds, VOC, breath, asthma, COPD, diabetes, pneumonia, cystic fibrosis, *Staphylococcus aureus*

## Abstract

This work presents the development of tin oxide nanofibers (NFs) and nanoribbons (NRs) sensors with graphene as a dopant for the detection of volatile organic compounds (VOCs) corresponding to different chronic diseases (asthma, chronic obstructive pulmonary disease, cystic fibrosis or diabetes). This research aims to determine the ability of these sensors to differentiate between gas samples corresponding to healthy people and patients with a disease. The nanostructures were grown by electrospinning and deposited on silicon substrates with micro-heaters integrated. The morphology of NFs and NRs was characterized by Scanning Electron Microscopy (SEM). A gas line was assembled and programmed to measure a wide range of gases (ethanol, acetone, NO and CO) at different concentrations simulating human breath conditions. Measurements were made in the presence and absence of humidity to evaluate its effect. The sensors were able to differentiate between the concentrations corresponding to a healthy person and a patient with one of the selected diseases. These were sensitive to biomarkers such as acetone and ethanol at low operating temperatures (with responses above 35%). Furthermore, CO and NO response was at high temperatures (above 5%). The sensors had a rapid response, with times of 50 s and recovery periods of about 10 min.

## 1. Introduction

For centuries, the use of aromas was employed to identify different diseases through the breath. Doctors could identify diseases such as diabetes or kidney failure using this method based on experience [1]. Similarly, there are currently several applications where dogs are trained to differentiate among different odors. One of them is disease detection, where the animal can differentiate between a healthy person and a person with a particular disease, as they can smell volatile compounds at lower concentrations than humans [2,3].

The air exhaled by a person contains many VOCs, whose concentrations can vary depending on various external factors such as age, weight, sex or lifestyle. Diseases are another parameter that can modify the composition of the breath, in particular, some respiratory and digestive diseases [4].

Based on these ideas, electronic equipment capable of imitating olfactory organs of mammals began to be developed [5]. All this, together with the growth experienced in different areas such as chemical sensors, microelectronic designs, materials and artificial intelligence, has actively contributed to the technological development of medical applications. These devices are developed to prevent, diagnose and control different pathologies, which will increase life expectancy and, above all, the quality of life of people [6].

There was great growth in the gas sensors area with the extension and discovery of new nanomaterials and improved manufacturing techniques. This allowed obtaining more selective sensors towards different compounds and sensitivity to lower concentrations. We investigate graphene-doped tin oxide sensors responses as components of a home-made electronic nose in this work. Sensors based on sensitive one-dimensional nanostructured metal oxide layers were demonstrated to have superior performance in comparison to thin-film ones. Besides, tin oxide remains an essential material in the manufacture of resistive sensors. The use of graphene as a dopant in this type of sensor improves its electrical properties and increases the chemical sensitivity to different compounds [7,8].

Nanostructures can be synthesized through different methods such as chemical vapor deposition, electro-deposition, thermal evaporation, rapid oxidation, electrospray or electro-spinning. The latter is a simple and economical method for the manufacture of nanostructures. The fibers manufacturing process begins with using a high molecular weight organic polymer, incorporating a metallic precursor. The process consists of applying an electric field to this solution. The solution formed by the organic polymer and the metallic precursor has a specific viscosity. The solution is projected onto the surface when the electric field effect is greater than the surface tension [9,10,11].

Improvements in electronic noses based on nanostructured materials will positively influence the challenges medicine faces today, enabling the development of economic and portable equipment. These devices open the door to better diagnosis and more effective control of various chronic diseases, such as asthma or diabetes (affecting around 339 and 422 million people worldwide, respectively) [12,13,14]. This technology would also help control other serious diseases that affect the world population to a lesser extent, such as COPD and cystic fibrosis.

On the other hand, pneumonia or respiratory infections can be a significant problem for patients with these chronic diseases. Due to this, they are more likely to contract them and suffer an exacerbation of chronic disease symptoms. One of the most common infections is *Staphylococcus aureus*, which affected more than 119,000 people in the USA in 2017, with a mortality rate of about 17% [15]. Previous work has provided a comprehensive review of the biomarkers of respiratory diseases [16].

These pathologies cause an increase in the concentration of the corresponding compound in the patient breath, compared to a healthy person. Furthermore, this concentration increases with the worsening of the disease. Table 1 shows the concentration ranges for healthy and sick people for each biomarker used to detect diabetes (acetone), COPD, cystic fibrosis or asthma (NO and CO) [16].

Moreover, if the sample contains *Staphylococcus aureus*, certain compounds such as alcohols and aldehydes can be observed. The concentration of these compounds increases exponentially for hours as the bacterial concentration in the culture increases. Of these, ethanol was selected, one of the gases where most variation is observed between 1 and 6 h. Table 2 shows the evolution of ethanol concentration in cell culture over time.

This research aims to determine the capacity of these sensors to differentiate between gas samples (generated in the laboratory) corresponding to healthy people and patients with a disease. The final goal is to develop a multisensory system for this application.

## 2. Materials and Methods

### 2.1. Materials

Polyvinyl alcohol (PVA, Mw = 80,000 g/mol) and tin (IV) chloride pentahydrate (SnCl_4_·5H_2_O, 98%) precursor materials were supplied by Sigma-Aldrich (St. Louis, MO, USA). Distilled water as a solvent. Pristine graphene (PG) was used as a dopant prepared by the liquid phase exfoliation (method described in previous work [22]). The process is a sonication-assisted exfoliation of graphite flakes in a hydroalcoholic solution. An isopropanol dispersion was obtained at a concentration of 10^−1^ mg/mL. Raman analysis showed that this method exfoliates graphite down to less than five layers [22].

### 2.2. Preparation of Precursor Solution

First, the aqueous PVA solution (11 wt.%) was prepared by dissolving it in distilled water and heating it to 90 °C while stirring it for 45 min. Then 1.2 g of SnCl_4_·5H_2_O and 4.1 mL of graphene solution were added, obtaining a final concentration of 100 ppm (graphene/Sn 0.01 wt.%). Finally, the solution was cooled to room temperature and stirred during the process to obtain a homogeneous mixture and avoiding leaving bubbles that could spoil the electro-spinning process [23].

### 2.3. Synthesis of Tin Oxide Nanostructures with Pristine Graphene

An electrospinning process was used to synthesize SnO_2_-graphene nanofibers and nanoribbons. The PVA/SnCl_4_·5H_2_O/PG precursor solution was loaded into a syringe pump connected to a metallic needle. A positive voltage of 19 kV was applied between the needle tip and metal collector at 6 cm of distance. The micromachined silicon substrate was placed on the collector and the precursor solution was directly electrospun onto the substrate at a flow rate of 2 µL/min for 20 min. All sensors were prepared under the same conditions. Electrospinning system details were described in a previously published paper [23]. Subsequently, the electrospun samples were heated to 450 °C for 24 h to remove the polymer component and obtain a sensitive layer of SnO_2_-PG.

### 2.4. Gas Line Setup

**Figure 1:**
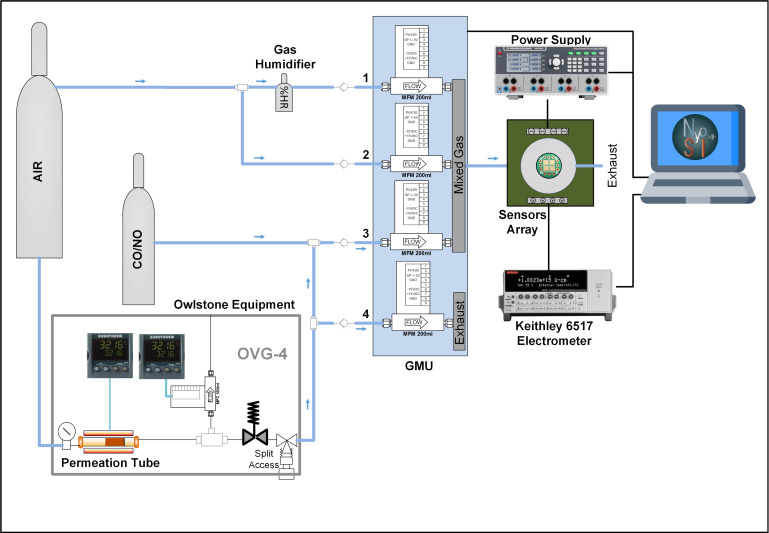
Measurement setup.

The measurement setup shown in Figure 1 was used to prepare the gas mixture and test the performance of the sensors. The measurements were carried out at a constant flow rate of 100 mL/min and a temperature range between 25 °C and 300 °C. The gases were generated from calibrated bottles of 10 ppm of CO and 200 ppb of NO (certified by Nippon Gases España S.L.U., Madrid, Spain), which were selected as the biomarkers of asthma, COPD and cystic fibrosis. On the other hand, ethanol and acetone permeation tubes were used to generate the concentration of the selected compounds *Staphylococcus aureus* infection and diabetes, respectively. Both permeation tubes were home-developed and subsequently calibrated for use. Table 3 shows the measured concentrations for each of the biomarkers used in this work.

The permeation tube was placed in the oven of the OVG-4 vapor generator module (Owlstone, Cambridge, UK) to generate ethanol or acetone concentration. The oven temperature was placed at the necessary value to achieve the desired concentration knowing the permeation rate, calculated by calibration. The oven flow rate was set at 100 mL/min, thus obtaining the maximum concentration required and then diluting with air afterward. It was necessary to ensure that the oven conditions remain constant throughout the measurement process.

A humidity generator was also incorporated to prepare gas mixtures more similar to human breath. The exhaled air can contain between 90 and 95% relative humidity (RH). The experiments were carried out without humidity and at 50% RH to determine its influence on sensor response.

A gas mixing unit (GMU, Ray IE, Cáceres, Spain), with four mass flowmeters, was used to control the gas mixture concentration. Flowmeter no. 1 controls the humidifier airflow, while flowmeter no. 2 controls the dry air. When a calibrated gas bottle is used, flowmeter no. 3 controls the CO or NO bottle flow.

On the other hand, when permeation tubes are used, flowmeters no. 3 and 4 are utilized to keep a constant airflow through the oven. The sum of the flow of both flowmeters must always be the same to maintain the permeation conditions. The flowmeter no. 3 controls the airflow that is desired to add to the mixture. On the contrary, the rest of the air, controlled by flowmeter no. 4, is discarded. The gas mixture obtained was passed through the cell where the sensors are located. The sensors were connected to the programmable power supply (HM7044, Rohde & Schwarz, Munich, Germany) to supply the heaters. An electrometer (6517 model, Keithley, Cleveland, OH, USA) is used to measure the sensor resistance. The whole process and equipment were controlled through the pc, using software home-developed with LabVIEW.

### 2.5. Sensor Description

**Figure 2:**
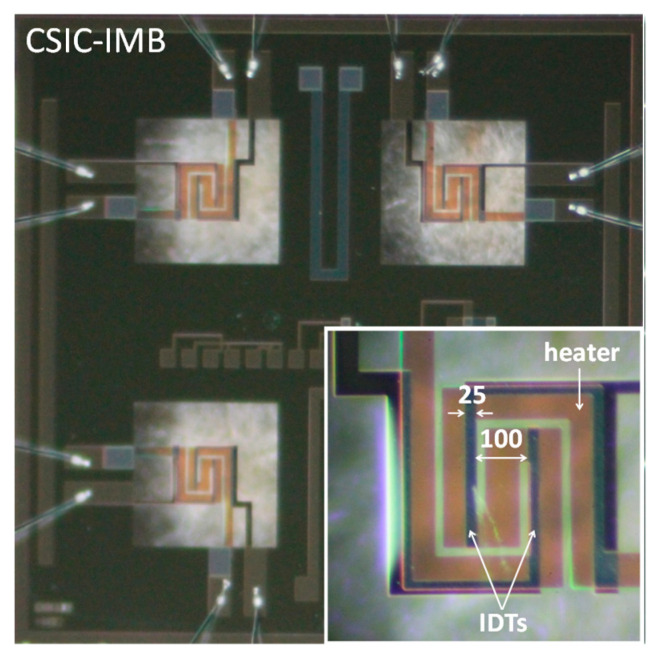
Silicon substrates with four microhotplates manufactured by CSIC-IMB. Insert (bottom right) show features of microhotplates: membrane, IDTs and heater (dimensions in µm).

The National Microelectronics Centre (CNM Barcelona, Spain) developed the micromachined silicon substrate used to deposit the nanostructures (Figure 2). This device incorporates four microhotplates with an active area of 400 × 400 µm. It has integrated microheaters that allow the sensitive layer to reach up to 500 °C and interdigitated electrodes (IDTs) to measure the sensitive layer resistance. The micromachined silicon substrate is mounted on a TO-8 package for subsequent characterization with the sensitive material [24]. This package is then placed inside the measuring (o test) cell, which has two holes (gas inlet/outlet) and is connected to the gas line.

## 3. Results and Discussion

### 3.1. Morphological Characterization

**Figure 3:**
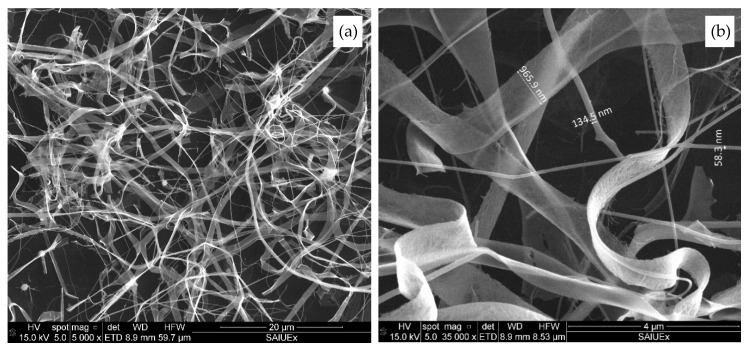
SEM images of tin oxide nanofibers and nanoribbons doped with PG after calcination at 450 °C. (**a**) Low and (**b**) high magnifications.

The surface morphology of the sensitive material (PG-SnO_2_) was characterized by scanning electron-microscopy (Quanta 3d FEG, FEI, Hillsboro, OR, USA). SEM images (Figure 3) reveal two types of nanostructures, NFs and NRs, though NRs seem predominant.

Figure 3a shows a random distribution of NFs, NRs and a multitude of nano-grains that form both nanostructures. NRs, nearly 1 μm in diameter, are very thin, so the nanostructures underneath can be visualized. In general, NFs have a diameter of around 50 nm, but some can be observed with a diameter between 130–150 nm (Figure 3b). Regarding their length, both NFs and NRs are very long, exceeding 20 μm in many cases. It should be noted that the PG was not appreciated in the SEM image due to the concentration in the material being low. However, the SnO_2_ NFs and the presence of graphene NSs was indirectly confirmed by elemental microanalysis conducted by energy dispersive X-ray spectroscopy (EDX). The spectrum ensures that the nanofibers contain O and Sn atoms. A carbon peak is due to the graphene loading, not detected in the pristine SnO_2_ NFs, as displayed in our previous work [23].

NFs and NRs are intertwined, forming non-uniform porous networks. The pore area of these networks varies from several microns to tenths of nanometers. Large pores with a high surface/volume ratio are favorable for gas detection facilitating gas penetration. Reports confirm that nanoribbons were obtained by electrospinning with PVA solutions at low concentrations (about 13%) and high Mw (50,000–89,000 g/mol) [25]; In_2_O_3_ NFs and NRs were also produced by this process [26]. Other works proved how NRs formation could be due to a small flow (lower than 2 L/min) in the electrospinning process [27,28].

### 3.2. Sensors Response

Different concentrations of acetone, ethanol, NO and CO were utilized to determine the sensor response. This response is calculated using Equation (1) for reducing gases, where Ra and R correspond to the sensor resistance under exposure to the air and selected gas, respectively:

Response = (R_a_ − R) × 100/R, (1)

**Figure 4:**
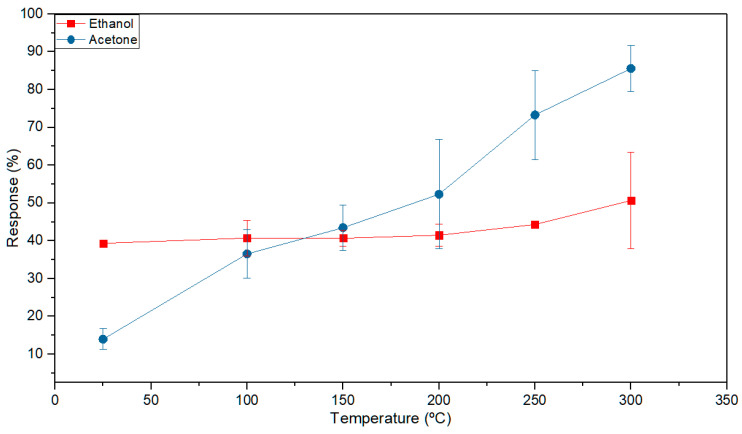
Sensor response 1 to 2 ppm ethanol and 4 ppm acetone at different operating temperatures in dry air (0% RH).

Detections were carried out in the range of 25 to 300 °C to determine the temperature for maximum sensor response. Ethanol and acetone were selected as the most representative gases to show their responses. This is very similar in all sensors, with around 16% variations demonstrating that the deposition process is uniform. Therefore, only one sensor response will be provided to simplify the graphs. The sensor response to 4 ppm acetone and 2 ppm ethanol at the different operating temperatures is shown in Figure 4. This increases with the operating temperature, reaching the maximum value at 300 °C (Figure 4).

**Figure 5:**
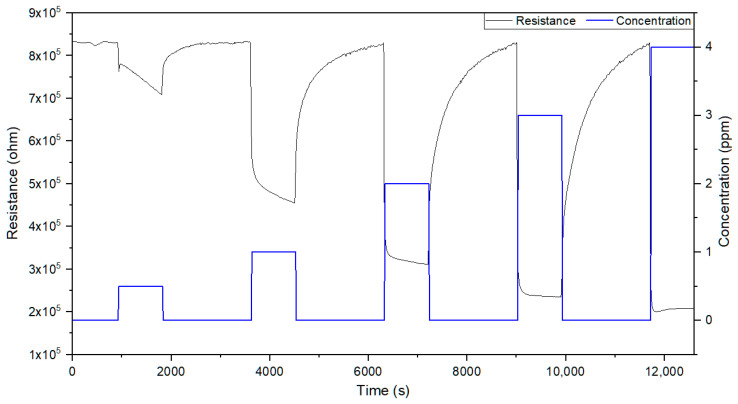
Resistance curve to different concentrations of acetone (0.5, 1, 2, 3 and 4 ppm) in dry air (0% RH) at 300 °C.

At 300 °C, as shown in the experimental detection curves (Figure 5), detection processes are fast with response times of 50 s and recovery periods of about 10 min. All sensors exhibited similar responses to the gases detected.

The measurements with different concentrations of the selected gases were made at 300 °C, as a more significant response is obtained at this temperature. On the following response graphs, the green shade represents the concentrations corresponding to healthy people, while the red shade corresponds to the concentrations of people with the pathology under study in each case. For ethanol, this type of representation is not used as it shows the evolution of the infection in the culture over time.

**Figure 6:**
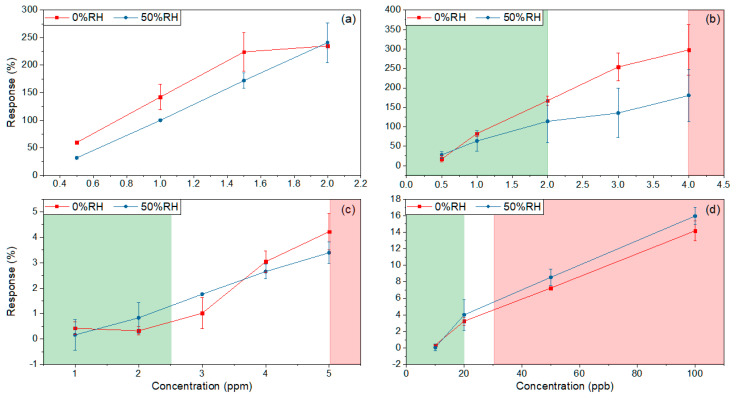
Responses at 300 °C in dry (0% RH) and humidity (50% RH) air to different concentrations of biomarkers: (**a**) ethanol; (**b**) acetone; (**c**) CO; (**d**) NO. The green and red shades represent the concentrations corresponding to healthy and sick people, respectively.

Detections were carried out in dry (0% RH) and wet (50% RH) air (Figure 6) to evaluate the effect of humidity at 300 °C (higher response temperature). The response of the sensors in the presence of humidity depends on the gas detected. In wet atmospheres, the sensors have a lower response to VOCs such as acetone and ethanol, while for CO and NO, this response is slightly higher.

The percentage variations between the highest concentration in the healthy person and the lowest in the patient (Table 1) with different diseases are presented in Table 4. For acetone and NO, few variations were observed between the measures carried out with 0% RH and 50% RH.

Finally, ethanol was measured at different concentrations as a biomarker of *Staphylococcus aureus* infection. Figure 6a shows the variation of the response when the proliferation of this bacterium increases in cell culture, generating a more significant ethanol amount. As shown in Table 5, there is a more relevant variation between the lowest and highest concentration in the measures carried out at 50% RH than at 0% RH.

**Figure 7:**
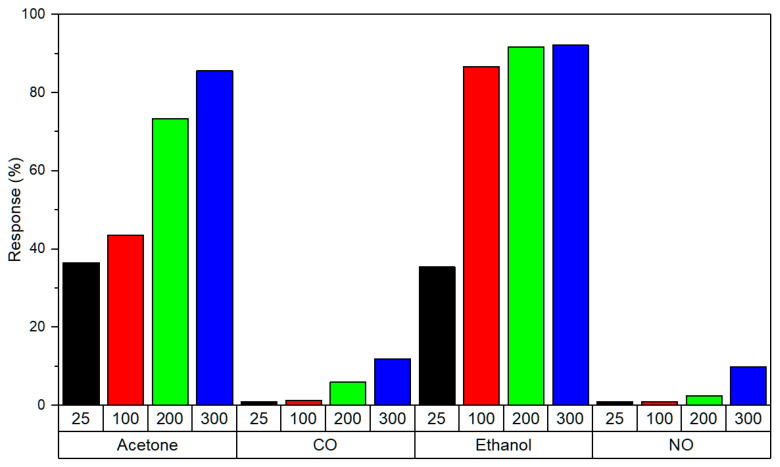
Sensor responses to 2 ppm ethanol, 4 ppm acetone, 5 ppm CO, and 100 ppb NO at different operating temperatures (25, 100, 200, and 300 °C).

The sensors present different responses to the target gases in the employed temperature range. Figure 7 compares the responses to the different gases used (2 ppm ethanol, 4 ppm acetone, 5 ppm CO and 100 ppb NO). The highest responses are obtained for acetone and ethanol for all ranges of temperatures, with responses above 35%. However, responses to CO and NO are minimal at low temperatures and begin to be appreciable above 100 °C. Therefore, distinct responses will be obtained for each biomarker when each sensor has a different operating temperature.

The gas detection mechanism on semiconductor metal oxide is generally described based on the “ionosorption model” [29,30]. When tin oxide (n-type semiconductor) is exposed to a reducing gas such as the gases detected in this work (CO, ethanol, acetone), the adsorbed oxygen species will react with the reducing gas releasing the trapped electrons and therefore reducing the resistance of the sensor. According to the ionosorption model at 300 °C, oxygen is ionosorbed as O^−^ [31]; the simplified reactions that can take place on the surface of the tin oxide during the detection of the reducing gases tested are as follows [32,33,34]:

Initial in air: O_2_ (g) + e^−^ → O^−^ (ads), (2)

For ethanol: C_2_H_5_OH (g) + 6O^−^ (ads) → 2CO_2_ (g) + 3H_2_O (g) + 6e^−^, (3)

For acetone: CH_3_COCH_3_ (g) + 8O^−^ (ads) → 3CO_2_ (g) + 3H_2_O (g) + 8e^−^, (4)

For CO: CO (g) + O^−^ (ads) → CO_2_ (g) + e^−^, (5)

It should be remarked that NO behaves as a reducing gas in the tested temperature range (25–300 °C). All the detection processes proved that the behavior and resistance of all sensors decreased in the presence of NO. Accordingly, a similar reaction to the reducing gases previously mentioned can be considered for NO (6):

NO (g) + O^−^ (ads) → NO_2_ (g) + e^−^, (6)

Nitrogen monoxide is unstable and oxidizes to form NO_2_ (oxidizing gas). However, detections were performed to confirm that NO does not oxidize in the test cell, which could be due to the optimal design of the gas line, facilitating immediate access to the gas.

In general, the detections in the wet atmosphere carried out in this work demonstrated that the sensor responses to VOCs (acetone and ethanol) decrease in the presence of water vapor. Water molecules can be adsorbed by physisorption (molecular water) and chemisorption (hydroxyl groups), although at temperatures above 200 °C, molecular water is no longer present on the surface. Adsorption of water vapor produces an increase in the electronic conductivity of tin oxide. This is due to the chemisorbed hydroxyl groups being bound to tin atoms and the proton, H^+^, is ready for the reaction with lattice oxygen or adsorbed oxygen [24,35]. The effect of humidity on the response to gases is not established. One of the most accepted hypotheses is that water displaces the chemisorbed oxygen.

Moreover, water molecules also act as a barrier. The VOCs detection scenario would be that water vapor inhibits oxygen adsorption on the tin oxide surface. However, in CO and NO detection, a response increase is observed: The higher response is due to two reactions that can take place simultaneously with the adsorbed species (O^−^ and OH^−^). It was demonstrated that CO reacts with the OH^−^ ions adsorbed on the surface of the semiconductors to form anions (HCOO_2_)^−^ as intermediate species according to the reaction [36]:

CO (g) + OH^−^ (ads) → H^+^ (ad) + CO_2_ (g) + 2e^−^, (7)

Finally, it should be noted that in this work, the presence of graphene improves the detection at low temperatures and increases the response to gases, possibly due to n–p heterojunction. In our previous work, undoped tin oxide nanostructures, also prepared by electrospinning, did not obtain a significant response to acetone below 300 °C [23]. Interactions between graphene nanosheets (NSs) and tin oxide NFs/NRs are important to understand the electrical transport properties and sensing mechanism of the device based on graphene NS-loaded SnO_2_ NRs/NFs. Graphene NSs and SnO_2_ NRs/NFs form a heterojunction. SnO_2_ and graphene work functions are 4.55 and 4.60 eV, respectively [37,38,39,40], and the Fermi level of SnO_2_ lies higher than the Fermi level of graphene.

Consequently, to equilibrate the Fermi level, electrons flow from SnO_2_ to graphene, causing a depletion region on the SnO_2_ surface, resulting in increased base resistance. Specifically, at room temperature, the base resistance of SnO_2_ NRs/NFs is in the tens of KΩ range. The addition of 100 ppm of graphene results in a slight increase in initial resistance. This will form potential barriers at the heterojunctions that will be modulated by gas adsorption. This mechanism would add to the SnO_2_ intergrain barrier modulation and could explain the increase of sensor response. The morphology of the sensor layer with NFs and NRs forms porous networks facilitating gas penetration and improving sensor sensitivity.

## 4. Conclusions

The above results confirm that graphene-doped tin oxide sensors can be used in an electronic nose for disease detection and control applications. The PG-doping effect seems to enhance the detection at low temperatures, compared to previous publications without PG, and increase the response at low gas concentrations. On the one hand, this will allow using sensors at different temperatures to differentiate between different gases and detect lower concentrations. Moreover, on the other hand, it will be possible to use sensors without heating resistances that will allow simplifying the substrate and making a more energy-efficient device. All this will contribute to the development of portable and low-cost equipment that could be used in health centers or by patients themselves at home. For all these reasons, we consider that this technology has a great potential to become a wearable technology, gadget or even to be integrated into smartphones or smartwatches.

The sensors have detected the gases selected as biomarkers (acetone, CO, ethanol and NO) in the concentrations corresponding to healthy people and patients with different pathologies. The highest response was obtained at 300 °C, although the sensor also responded to lower temperatures, particularly at room temperature for acetone and ethanol. Different responses can be obtained with similar sensors, heating each of them to a different temperature to detect the biomarkers selected. This device can. Therefore, be used in a multi-sensorial system (electronic nose). It was also observed that humidity does not prevent adequate gas detection, but it does modify the sensor responses. The absorbed OH^−^ groups can displace part of the adsorbed oxygen species, decreasing the sensor response. This is observed in the detection of ethanol and acetone. However, both interact with absorbed species (OH^−^ and O^−^) in CO and NO detection, increasing the sensor response.

We will consider using higher percentages of relative humidity to determine its effect on sensor response in future work. In this way, we will approximate the values found in the breath of a person. We will also use non-heated substrates to achieve a more energy-efficient device with longer battery life. Other metal oxides could be used as a sensitive layer and different dopants to achieve better selectivity and a broad spectrum of response when used in a sensor array in an electronic nose.

## Figures and Tables

**Table 1:** Concentration ranges are used for the different biomarkers, both for healthy people and for patients with different pathologies.

Disease	Biomarker	Concentration Range for Healthy Persons	Concentration Range for Ill Persons
Diabetes [17]	Acetone	0.5–2 ppm	>4 ppm
Asthma, COPD and Cystic Fibrosis [16,18,19,20]	NO	1–20 ppb	>25 ppb
	CO	1–2 ppm	>5 ppm

**Table 2:** Evolution of ethanol concentration in *Staphylococcus aureus* cell culture over time.

Disease	Biomarker	1.5 h	3 h	4.5 h	6 h
*Staphylococcus aureus* infection [21]	Ethanol	89 ppb	237 ppb	6173 ppb	11,695 ppb

**Table 3:** Measured concentrations for each biomarker.

Biomarker	Concentrations
Ethanol	0.5, 1, 1.5 and 2 ppm
Acetone	0.5, 1, 2, 3, and 4 ppm
CO	1, 2, 3, 4 and 5 ppm
NO	10, 20, 50 and 100 ppb

**Table 4:** Percentage variation between the maximum concentration in a healthy person and the minimum in a sick person, both at 0% RH and 50% RH.

Biomarker	0% RH	50% RH
Acetone	130.5%	66.3%
CO	3.9%	2.6%
NO	4.1%	4.5%

**Table 5:** Percentage variation between 0.5 and 2 ppm of ethanol, both at 0% RH and 50% RH.

Biomarker	0% RH	50% RH
Ethanol	175.1%	209.1%
